# The diversity of interest in later-life entrepreneurship: Results from a nationally representative survey of Americans aged 50 to 70

**DOI:** 10.1371/journal.pone.0217971

**Published:** 2019-06-05

**Authors:** Cal J. Halvorsen, Yu-Chih Chen

**Affiliations:** 1 School of Social Work, Boston College, Chestnut Hill, Massachusetts, United States of America; 2 Brown School of Social Work, Washington University in St. Louis, St. Louis, Missouri, United States of America; Jinan University, CHINA

## Abstract

While older entrepreneurs are more likely to be male, white, and have higher levels of human, social, and financial capital, we know less about interest in later-life entrepreneurship. This study estimates entrepreneurial interest in a nationally representative sample of Americans aged 50 to 70 using partial proportional odds modeling. We estimate that more than 31 million older Americans have some interest in entrepreneurship and reveal key predictors of this interest (e.g., younger age). Importantly, the findings indicate that a more diverse group of older adults are interested in entrepreneurship than have become entrepreneurs, suggesting the need for additional research on the potential disparities between entrepreneurial interest and action in later life.

## Introduction

While the scholarship on entrepreneurship in later life is growing, little research has been conducted on *interest* in entrepreneurship among older adults, an important precursor to entrepreneurial intentions and activity that may be less constrained by real or perceived barriers and opportunities than by intentions or activity themselves. To date, academic scholarship on interest in entrepreneurship in later life is scant, with the exception of a one-page research brief on the subject (see [1]).

Understanding who is interested in entrepreneurship in later life has been identified as an area in need of additional research [2]. Findings from this research have important implications for scholars and practitioners focused on increasing entrepreneurial opportunities and financial security in later life. If certain segments of older adults are found to have higher levels of entrepreneurial interest but lower levels of activity, for example, programs and policies could target those groups to help them successfully achieve these aspirations, thereby increasing the pool of potential entrepreneurs. This is important for individuals and the economy, as entrepreneurship can provide workplace flexibility with continued income for older adults [3] while boosting the economy through startup activity [4].

Scholars have suggested that older adults may be more successful as entrepreneurs because of their higher levels of human, social, and financial capital, on average, than younger people [2,5]. These advantages, built up over the course of one’s life, make it easier to access financing for business startup and tap into one’s personal and professional networks for business growth. Recent empirical scholarship has supported this claim, revealing that the mean age of entrepreneurs in the U.S. who hired at least one employee was 41.9 years between 2007 and 2014, while the mean age of founders in the top 1,000 highest growth ventures was 45.0 [6]. These findings highlight the economic benefits of mid- and later-life entrepreneurship while contesting the stereotype that entrepreneurship is the realm of the young.

This paper contributes to our empirical and theoretical knowledge of entrepreneurial interest in later life by analyzing data from a novel sample of older Americans. This study has two aims: first, to estimate the level of interest in entrepreneurship among older Americans; and second, to assess what factors are associated with this interest. We begin by discussing the ways entrepreneurship has been defined, what is known about entrepreneurship in later life, and factors that are associated with later-life entrepreneurship and the theoretical case for why these relationships may exist. We then describe this paper’s specific study, outline our results, and discuss the program, policy, and research implications of these findings. Although scholarship on later-life entrepreneurship is growing, our study is exploratory in nature due to the lack of scholarship examining *interest* in entrepreneurship in later life; therefore, hypotheses are not provided.

### Definitions of entrepreneurship

The terms self-employment and entrepreneurship are often used interchangeably, as is the case in several of the references in this paper. However, the term entrepreneurship is frequently used in more conceptual and idealistic ways. For example, some definitions focus on the act of innovation and the pursuit of opportunities as integral aspects of entrepreneurship (e.g., [7,8]); they focus less on starting a new organization, which is the working definition in this paper. Although these definitions are appropriate for several purposes, including case studies and theoretical development, they are difficult to operationalize for applied research purposes [2,5]. In line with this logic, neither the Current Population Survey, which is often used to measure rates of entrepreneurship among the entire adult population (e.g., [9]); the Health and Retirement Study, which is often used to measure entrepreneurship among older adults (e.g., [10]); nor other major surveys regarding entrepreneurship (e.g., the Global Entrepreneurship Monitor) operationalize the term with these subjective concepts. Instead, they ask if respondents are self-employed, work for themselves, or own businesses.

Scholars have often categorized entrepreneurs into different types. Weller and colleagues defined entrepreneurs as those who own and manage a business that is worth more than $5,000 and independent contractors as those who own and manage a business worth less than $5,000 [11,12]. Others differentiate between those who have employees and those who do not [13,14]. Still, others differentiate between necessity- and opportunity-based entrepreneurship, with those who transition from unemployment considered necessity entrepreneurs and those who transition from other labor force statuses called opportunity entrepreneurs [9]. There are differences between these groups in relation to motivations, experiences, and outcomes. For example, a pan-European study found that entrepreneurs with employees had longer business survival rates than those without employees [14]. Using an innovation lens on entrepreneurship—often called Schumpeterian or novel entrepreneurship—scholars have argued that these differences matter. Specifically, they argue that Schumpeterian entrepreneurship promotes the most economic growth and that our public policies should focus on promoting it [15,16].

Regarding older entrepreneurs, scholars have used different terms. These include encore entrepreneurs (e.g., [1,17]), grey entrepreneurs (e.g., [5]), and seniorpreneurs [18], among others. We simply use the terms self-employed or entrepreneur, modified by an age descriptor (e.g., older adult entrepreneurship or self-employment in later life).

### Entrepreneurship and self-employment in later life

Self-employment is a major form of work among older adults. First, the percentage of Americans *in the workforce* who are self-employed rises with age, from a rate of 7.2% for Americans aged 16 to 49 in 2014 to 15.8% of adults aged 60 to 64 and 30.2% of adults 75–79 [19]. Second, the percentage of *all* Americans, aged 62 and older, who were self-employed increased from 4.2% in 1988 to 5.4% in 2015 [20]. And third, looking at four age groups between 20 and 64 from 1996 to 2016, Americans between the ages of 55 and 64 consistently had some of the highest rates of new self-employment activity and made up nearly one-quarter (25.5%) of new entrepreneurs in 2016 [9]. Similar rates exist in Europe. Using data from 11 countries in the Survey of Health, Ageing, and Retirement in Europe, for example, scholars have shown that the rates of self-employment increase with age among those who remain in the workforce [21]. Further, more than two in five of those who are self-employed in several European countries, including Germany, Sweden, and the United Kingdom, are aged 50 and older [22]. In the U.S., the increasing numbers of older adults, predicted rise in labor force participation rates [23], and expansion of health care access that is not tied to one’s employer [24] all point to an increasing prominence of this form of work at this stage in life.

Program designers and policymakers have noticed this trend. In 2014, the U.S. Senate Special Committee on Aging and the Small Business and Entrepreneurship Committee held a joint session on “the challenges and advantages of senior entrepreneurship” [25]. Additionally, in-person and online trainings and workshops devoted to encouraging and helping older adults start new businesses are proliferating from the national level (e.g., programs by AARP and the Small Business Administration) to the local level (e.g., workshops by the St. Louis, Missouri license collector’s office and the Florida nonprofit organization, Encore Tampa Bay).

Scholarship on later-life entrepreneurship is still in its nascent stages. While studies have documented the correlates of moving into this type of work (e.g., [10,12]), the potential increase in quality of life from moving into this work [26], and the different types of self-employment in later life [27], we could only identify one research brief that documented *interest* in later-life entrepreneurship specifically among older adults. Reporting results from a nationally-representative telephone survey of 1,000 Americans aged 44 to 70—a precursor to the survey analyzed for this article—this study found that one in four respondents had a high level of interest in becoming an entrepreneur [1]. The relationship between entrepreneurial interest and factors such as gender, race, education, and income, however, was not elucidated. Given the lack of empirical and theoretical scholarship on key groups of older adults and interest in entrepreneurship, it is difficult to enact targeted program and policy interventions to increase access to entrepreneurial opportunities, highlighting a need for foundational, descriptive research.

### Conceptual framework and supporting scholarship

**Fig 1:**
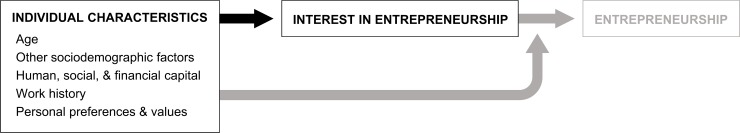
Conceptual model of entrepreneurial interest in later life. Adapted from Halvorsen & Morrow-Howell. [2]. Conceptual framework on self-employment in later life: Toward a research agenda. *Work*, *Aging & Retirement*, *3*(4), 313–324, by permission of Oxford University Press.

This article is guided by an adapted version of the conceptual framework put forth by Halvorsen and Morrow-Howell [2], as shown in Fig 1. The original framework, developed after a review of the literature, argued that a set of individual and contextual antecedents lead to the self-employment experience, which in turn leads to a set of individual and societal outcomes. In our framework, which was adapted to consider interest in later-life entrepreneurship, we identify potential antecedent factors to entrepreneurship in later life. For this study, we examined the relationship between individual characteristics and entrepreneurial interest. Antecedents include age and other sociodemographic factors (e.g., gender); human capital (e.g., education), social capital (e.g., marital status), and financial capital (e.g., total household wealth); labor force status; and personal preferences and values (e.g., meaning of work). It is important to note that while this study is guided by a conceptual framework developed from empirical and theoretical scholarship on *work and entrepreneurial activity* in later life, this study is pre-theoretical in that it seeks to find factors related to *interest* in later-life entrepreneurship that can be replicated in future work while contributing to the development of future theoretical perspectives, programs, and policies [28]. As such, this exploratory study aims to examine the relationship between entrepreneurial interest and key factors related to human, social, and financial capital, as well as individual preferences and values. While we propose that human, social, and financial capital are *not* positively related to interest in later-life entrepreneurship, echoing Proposition 4b by Halvorsen and Morrow-Howell [2], we offer no hypotheses related to specific variables.

#### Sociodemographic factors

Self-employment among those in the labor force has been shown to rise with age [19]. This is counter to a general negative association with age in the desirability to become self-employed. For example, scholars who examined responses from nearly 14,000 individuals in 21 countries who were not self-employed revealed an inverse U shape relationship between desirability and age, with a peak in desirability around the age of 22 [29]. Yet considering responses from more than 2,500 respondents aged 18 to 64 who were *already* engaged in some type of entrepreneurial activity, entrepreneurship was positively associated with age among those who preferred to have no employees while it had an inverse U relationship—peaking in the late 40s—among those who wanted to hire employees [30]. As such, the type of self-employment may make a meaningful contribution to discussions on self-employment aspirations in later life.

Considering gender, it has been widely established that women are less likely to start new businesses than men (e.g., [31]). Using the 2014 Annual Survey of Entrepreneurs, Tareque [32] found that in all ages, men are more likely to become entrepreneurs, with male-owned firms comprising nearly two in three (64.5%), female-owned firms comprising about one in five (20.5%), and jointly-owned firms comprising about one in seven (15.0%) of all firms. Looking at *new* entrepreneurs in 2016, data from the Current Population Survey showed similar results, with about three in five (60.5%) new entrepreneurs identifying as male, compared to two in five (39.5%) identifying as female [9]. While older entrepreneurs are also more likely to be male, the gender imbalance decreases slightly when considering those who transitioned into entrepreneurship after the age of 50. For example, Zissimopoulos and Karoly [13] found that less than one third (31.8%) of adults who became self-employed before the age of 50 were female, compared to about two in five (39.0%) who became self-employed after the age of 50. Some scholarship has examined entrepreneurial intentions by gender, finding mixed results regarding its ability to predict interest [33–35]. However, these studies analyzed data from college or university students enrolled in business courses, making it difficult to extrapolate the findings to the socioeconomically diverse older population.

Considering race, rates of startup activity among Americans ages 20 to 64 have been higher in white than black Americans since at least 1996, and for every year since 1999, Latino Americans have had higher rates of startup activity than white Americans [9]. This study also revealed that the share of those who transitioned into self-employment from unemployment—suggesting necessity instead of opportunity—has been consistently higher among black and Latino Americans than white Americans. While less research has been conducted on self-employment and race among older adults, one study of Americans aged 51 to 67 found that there was no difference between the likelihood of transitioning from fulltime wage-and-salary work to self-employment by racial group [36].

The original conceptual framework proposed by Halvorsen and Morrow-Howell included work history [2], arguing that older adults may feel pushed or pulled into entrepreneurship due to their unique work experiences. Scholars have considered unemployment, for example, in relation to future entrepreneurship [37,38], with transitions to self-employment from unemployment increasing during the Great Recession among Americans [38]. Other labor force statuses have also been considered, including transitions to self-employment from wage-and-salary work, disability, and retirement [10,36]. For our study, we use labor force status at the time the survey was conducted to assess how working for someone else and being self-employed, retired, or disabled relate to interest in future entrepreneurial endeavors.

Further, scholars have discussed the importance of self-employment in both rural and urban settings. While rates of self-employment among Americans aged 15 and older in rural settings have historically been higher than in suburban and central city locations, rural self-employment rates have declined more sharply over the past three decades with central city self-employment rates slightly increasing [39]. This has been partly attributed to the overall decline in the population living in rural areas, many of whom may be seeking to advance their careers in urban areas [39].

#### Human, social, and financial capital

Scholars have found that older entrepreneurs, on average, have higher levels of human, social, and financial capital, and are more likely to come from historically advantaged groups. Human capital can be envisioned as assets within an individual, such as education and health [40], as well as one’s work experience. Social capital is associated with the connections between people, such as marriage and the strength of one’s support system [41]. And financial capital in the individual perspective can be measured by wealth and income [41]. Advantages in building these sources of capital have disproportionately been provided to key segments of the population, such as those who are identified as white and male. As such, studies have shown that older entrepreneurs are more likely to be male, white, and married, as well as have more formal education and higher incomes, than the general population [12,13]. Scholars have also found that those who are married have longer self-employment durations than those who are not [42], indicating the presence of a safety net through a spousal experience, income, or other attributes.

The relative advantage that some older adults hold may be what makes it possible to pursue later-life entrepreneurship. Weller and colleagues [12] found, for example, that older adult entrepreneurs are increasingly relying on their personal wealth as collateral; as such, those with fewer assets may be unable to pursue entrepreneurial activities, further increasing economic disparities in later life. Further, previous self-employment experience—a form of human capital—was found to be an important predictor of transitioning to new self-employment in later life [10,43]. Assessing the prior jobs of Americans aged 51 and older, Zissimopoulos and Karoly [10] found that previous self-employment experience among those who were unemployed, disabled, or retired was linked to more than a 30 percent increase in the likelihood of becoming self-employed two years later, in comparison to those without this experience. Previous self-employment experience may lead to increased knowledge of how to navigate the formation of new businesses as well as serve as an indicator of a long-running entrepreneurial interest. While having work-limiting health conditions has also been associated with transitions to later-life self-employment [36], it generally appears that a select and more advantaged group of older adults are doing so at greater rates.

To date, we are not aware of empirical scholarship linking interest in entrepreneurship and human, social, and financial capital factors among older adults. However, scholars have proposed that these capital factors may *not* be related to interest, emphasizing the universal nature of entrepreneurial interest and noting the possible existence of an “opportunity gap” due to less advantaged older adults who are interested in turning to entrepreneurship being less able to overcome real and perceived hurdles to startup [2]. Köllinger and Minniti [44], for example, documented an opportunity gap between white and black Americans when considering entrepreneurial activity.

#### Personal preferences and values

The conceptual framework also posits that the personal preferences and values of older adults are important factors leading to entrepreneurship. Mor-Barak [45] proposed that four key dimensions exist in relation to the meaning of work in later life: social, or the need to engage with people at work; personal, or the need to feel a personal sense of satisfaction and pride in one’s work; financial, or the need for income and benefits; and generativity, or the desire to leave a legacy for future generations. A study of German retirees found evidence that while the social, personal, and financial dimensions were related to post-retirement employment, generativity was not [46]. However, generativity—first described by Erik Erikson [47]—has been found to be an important factor among older entrepreneurs, having a positive relationship with reporting a family business successor and fully mediating the relationship between age and family succession [48]. Our study tests if these four dimensions are related to interest in entrepreneurship among older adults. Another study considered the potential impact of entrepreneurship among a group of interested older entrepreneurs [1]. It estimated that nearly half (48%) of aspiring entrepreneurs reported that having a positive social impact, meeting a need in their communities, meeting a social challenge, or providing a service to others would be the primary purpose for starting the new organization, in contrast to more profit- and autonomy-driven motivations.

The conceptual framework by Halvorsen and Morrow-Howell [2] also included factors such as perceived future time, personality, and risk tolerance, that may also be linked to interest in later-life entrepreneurship. These are worthy of future study yet, unfortunately, were not variables available in the data set used for this study. For a more in-depth discussion of these factors in relation to entrepreneurship, see Halvorsen and Morrow-Howell [2].

In this study, we aim to document interest in entrepreneurship among a group of people often forgotten by entrepreneurship scholars: Adults past midlife and into later life. This group, which has some of the highest rates of entrepreneurship, may very well present an even greater, untapped pool of talent. This information could lead to scholarship on potential gaps between interest and action, why certain older adults choose not to pursue—or are not successful in pursuing—entrepreneurial ventures, and where information, assistance, funding, and new programming or policies are needed.

## Methods

### Sample

Data were drawn from the Encore Career Survey, a novel study conducted by the national nonprofit research and advocacy organization, Encore.org, and the survey and market research firm, Penn Schoen Berland, in 2014. It aimed to understand the level of interest in different forms of work past midlife and was a follow-up to cross-sectional studies conducted in 2011, 2008, and 2005 (see [49–51]). Specifically, this study surveyed 1,000 Americans between the ages of 50 and 70 and included quotas to ensure that the sample was representative of the U.S. population by age, gender, and U.S. Census region.

The sampling frame consisted of online older adults obtained from the company, Critical Mix, and its panel of more than 1 million individuals who have undergone checks for accuracy that include screening for duplicate accounts and verification against known consumer databases. Online panel studies, which utilize sampling frames of adults who are selected for surveys based on their demographic information and answers to specific questions relevant to the study at hand, have become more common in recent years due to their convenience, the ability to quickly gather large datasets, and their lower cost compared to random-digit-dialed telephone surveys [52]. Although older adults have historically had lower rates of internet access, these rates are quickly changing, especially for the “young old” age group represented in this study [53].

As this data was provided to the authors in an anonymized format, the Human Research Protections Office at Washington University in St. Louis, where the bulk of the analysis was conducted, determined that this study was exempt from ethical review.

### Measurement

#### Interest in entrepreneurship

Respondents were asked the question, “How interested would you be in starting your own business or nonprofit venture in the next 5 to 10 years?” and answered using a 4-point Likert-type scale that was reverse coded for easier interpretation in this study’s analysis. Answer options range from 1 = “Not at all interested” to 4 = “Very interested”. Respondents could also indicate, “Don’t know.” We examined possible differences between the “Don’t know” group (*n* = 44) and those who indicated their level of entrepreneurial interest (*n* = 956) on this study’s explanatory variables, with few differences occurring; therefore, we excluded these respondents from the analyses.

#### Explanatory variables

Considering the links between a range of sociodemographic and human, social, and financial capital factors and later-life entrepreneurship (e.g., [12,13]), we tested similar factors on *interest* in entrepreneurship in later life. Sociodemographic factors include age (continuous), gender (1 = female, 0 = male), race (white, not Hispanic; black, not Hispanic; and all other races), urbanicity (1 = small town or rural area, 0 = city or suburb), and work status (working for pay, self-employed, retired, disabled, unemployed, and all other statuses).

Human capital was assessed using three variables, including educational attainment (four categories, from high school or less to master’s degree or higher), self-rated health status (reverse coded so that 1 = “Poor” and 5 = “Excellent”), and having completed adult education or training courses (1 = yes, 0 = no). Social capital was assessed using two variables, including marital status (1 = married, 0 = not married) and conducting volunteer work (1 = a few times per year or more, 0 = less than a few times per year). Our operationalization of social capital follows the individual-focused usage that is common in the literature on working and volunteering in later life (e.g., [54–56]). However, a deep body of scholarship has operationalized social capital as factors related to social norms, reciprocity, trust, the structure of relationships between and among actors, and neighborhood cohesion, among other factors (e.g., [41,57]) that may also play an important role in relation to self-employment in later life. Finally, financial capital was assessed using two variables, including total household income (five categories, from less than $20,000 to $125,000 or more) and total household assets, excluding real estate and other property (six categories, from less than $10,000 to $500,000 or more). Regarding this final variable, the survey our data are derived from did not ask about real estate equity; as such, we cannot consider this factor in our analysis. However, while real estate equity has been shown to be positively associated with entry into entrepreneurship [58], the magnitude of this relationship once incorporating all assets and debts has been shown to be relatively minor except for those in the top five percent of the wealth distribution [59].

To explore how personal preferences and values are associated with interest in entrepreneurship in later life, we tested five variables. The 16-item Meaning of Work Scale assessed four dimensions found to be important indicators in the meaning of work in later life [45], including personal (five items, α = 0.91), social contact (five items, α = 0.85), financial (three items, α = 0.75), and generativity (three items, α = 0.91). Reverse coded for ease of interpretation, answer options on the five-item Likert-type scale ranged from 1 = “Strongly disagree” to 5 = “Strongly agree.” Sample items include that paid work “gives me personal satisfaction” and “allows me to pass my knowledge to the next generation.” (For the complete scale, see [45].) Finally, respondents were asked what the primary reason would be for starting their organizations, with answer options including to “make money,” “work for myself,” “meet a social challenge,” “help others in need,” and “something else/don’t know.” (The survey combined the last category into a single question, preventing separate analyses of “something else” and “don’t know.”) Answers regarding meeting a social challenge and helping others in need—both acting as pro-social motivations—were combined to prevent low cell count issues during analysis.

### Analysis

We first conducted univariate and bivariate analyses to determine the level of interest in entrepreneurship among older adults as well as the key differences by level of interest in entrepreneurship on all factors used in the multivariable analyses. Given the ordinal character of the outcome variable, we present descriptive statistics by level of entrepreneurial interest, as well as unadjusted odds ratios using ordered logistic regression or partial proportional odds modeling, explained further below.

For multivariable models, ordered logistic regression is one of the most common models used with an ordinal outcome variable, as it accounts for the rank order of the data while not assuming equal differences between the possible values [60]. However, ordered logistic regression assumes that the coefficients for explanatory variables are equal in a series of cumulative logit models in which the response variable is recoded into a series of binary variables [61,62]. In other words, the coefficients should have the same relationship with the outcome variable, no matter how it is dichotomized (e.g., poor health compared to fair health and better, or good health or worse compared to very good health or better). The Brant test of coefficients [63] rejected the null hypothesis of equal coefficients for the entire model, indicating the need for further consideration: χ^2^ (52) = 80.87, *p* = .006. As such, we determined that using partial proportional odds models were of merit, as they allow for a subset of explanatory variables to include non-proportional odds (i.e., the parameters are different in a series of cumulative logit models, similar to multinomial logistic regression) while still allowing others to maintain proportional odds (e.g., the parameters are the same in a series of cumulative logit models, similar to a traditional ordinal logistic regression) [61,62,64]. In practice, this form of modeling can indicate meaningful differences in the coefficients of explanatory variables by level of the response variable. Peterson and Harrell [62], for example, illustrated the importance of modeling non-proportional odds by listing the odds of having higher severities of coronary artery disease by several explanatory variables. Following the guidance set forth by Williams [61,64], we used the *gologit2* user-written program in Stata to carefully consider the direction of the coefficients and their magnitudes, determining that a partial proportional odds model was necessary to allow for the coefficients on three variables—being self-employed, the personal meaning of work, and to meet a social challenge or to help others as the primary startup reason—to differ in the cumulative logit models. (The coefficients for the “all other races” variable, which is difficult to interpret, were also held constant.) Models will be displayed in table format following the example set by Craemer [65].

### Quality assurances

Several steps were taken in this study to ensure the quality of results. While the study was designed to be representative of the population of Americans aged 50 to 70 by age, gender, and U.S. Census region, we created survey weights to make the sample representative of the older population by race and education, in addition to the three previously mentioned categories. To do this, we employed the iterative “raking” procedure to benchmark our sample’s distribution to the distribution of the external population (see [66,67]) using publicly-available data from the U.S. Census Bureau for the year 2014 [68–71], the year this survey was completed, and the *survwgt* user-written program in Stata [72]. Additionally, variance inflation factor (VIF) tests showed no concern for multicollinearity (S1 Table), and tests for influential observations likewise raised no concerns. Due to this study’s exploratory nature, all tests of significance were two-tailed in nature with an alpha level of .05. We used Stata 15 for all analyses.

## Results

### Aim 1. Level of entrepreneurial interest among older Americans

A univariate analysis was conducted to estimate the percentage of Americans, aged 50 to 70, who reported interest in starting a business or nonprofit organization within the next five to 10 years. As Table 1 shows, about two in five respondents (38.8%) reported being at least somewhat interested, with about one in seven (14.0%) reporting the highest level of interest. About two in five (43.9%) reported no interest in starting an organization. Multiplying the findings by the more than 80 million Americans aged 50 to 70 in 2014 [68], this results in an estimated 31.1 million older Americans who were at least somewhat interested, and 11.2 million older Americans who reported the highest level of interest, in starting a new business or nonprofit organization in the next five to 10 years.

**Table 1:** Descriptive statistics of the level of interest in entrepreneurship among Americans aged 50 to 70.

	Very interested	Somewhat interested	Not too interested	Not at all interested
Interest in entrepreneurship	14.00%	24.75%	17.30%	43.94%
**Demographics**				
Age (50–70)	56.48 (0.53)	57.37 (0.40)	58.52 (0.52)	61.04 (0.35)
Gender: Male	56.40%	58.74%	54.95%	38.46%
Female	43.60%	41.26%	45.05%	61.54%
Race: White	56.82%	62.81%	80.40%	77.86%
Black, not Hispanic	21.45%	17.63%	10.74%	4.32%
All other races	21.73%	19.55%	8.86%	17.81%
Urbanicity: Urban	72.30%	65.10%	72.62%	64.97%
Rural	27.70%	34.90%	27.38%	35.03%
Work status: Working for pay	32.98%	39.36%	30.24%	23.75%
Self-employed	21.50%	9.39%	11.49%	5.47%
Retired	13.61%	19.33%	22.50%	42.31%
Disabled	17.17%	13.21%	15.89%	14.82%
Unemployed	8.99%	9.33%	12.07%	4.53%
Others	5.73%	9.38%	7.81%	9.12%
**Human capital**				
Education: High school or less	36.12%	33.99%	36.60%	49.48%
Associate’s degree	31.98%	28.44%	34.23%	22.67%
Bachelor’s degree	21.38%	21.81%	15.48%	16.43%
Master’s degree and above	10.52%	15.75%	13.69%	11.43%
Self-rated health (1–5)	3.25 (0.10)	3.16 (0.08)	3.08 (0.10)	3.01 (0.07)
Completed adult education/training	51.96%	44.37%	37.63%	36.30%
Did not complete	48.04%	55.63%	62.37%	63.70%
**Social capital**				
Married	50.71%	59.29%	54.87%	61.10%
Not married	49.29%	40.71%	45.13%	38.90%
Volunteer	51.64%	47.78%	37.64%	32.73%
Not a volunteer	48.36%	52.22%	62.36%	67.27%
**Financial capital**				
Income: Less than $20,000	18.61%	17.75%	18.86%	19.76%
$20,000 to $44,999	37.58%	31.11%	29.51%	34.28%
$45,000 to $72,499	22.30%	25.49%	23.72%	25.00%
$72,500 to $124,999	13.50%	15.64%	20.89%	14.93%
$125,000 or more	8.01%	10.01%	7.02%	6.03%
Assets: Less than $10,000	36.46%	39.04%	32.47%	41.51%
$10,000 to 49,999	23.32%	16.86%	18.22%	13.64%
$50,000 to $99,999	11.13%	10.88%	11.96%	10.17%
$100,000 to $199,999	7.66%	8.62%	9.32%	13.65%
$200,000 to $499,999	13.61%	13.78%	15.89%	10.49%
$500,000 or more	7.83%	10.82%	12.14%	10.54%
**Personal preferences and values**				
Meaning of work: Personal (5–25)	21.44 (0.33)	19.87 (0.36)	19.15 (0.27)	19.01 (0.26)
Social (5–25)	18.00 (0.50)	16.72 (0.34)	16.23 (0.27)	15.42 (0.26)
Financial (3–15)	11.26 (0.28)	10.68 (0.24)	10.35 (0.22)	10.16 (0.20)
Generativity (3–15)	11.92 (0.29)	11.23 (0.24)	10.82 (0.18)	10.37 (0.18)
Startup reason: Work for oneself	35.20%	24.95%	13.45%	10.15%
Make money	42.95%	40.60%	44.49%	37.13%
Meet social challenge, help others	21.00%	29.75%	23.47%	14.74%
Something else / Don’t know	0.85%	4.70%	18.59%	37.97%

*Note*. Results incorporate survey weights developed using the raking method in the *survwgt* program in Stata [72]. Means and standard deviations presented for continuous variables and column percentages presented for categorical variables.

### Aim 2. Predictors of interest in entrepreneurship in older Americans

Table 1 provides descriptive statistics for the sample by each category included in the multivariable analysis. Table 2 presents the bivariate proportional odds models for most variables; the variables for self-employment, personal meaning of work, and startup reason to meet a social challenge and help others show the partial proportional odds estimates, which are shaded in light blue to help in understanding.

**Table 2:** Bivariate proportional odds and partial proportional odds models for interest in entrepreneurship.

	uOR	SE	*p*	95% CI	
**Demographics**					
Age	.90	.01	< .001	.88	.92
Female (*ref*: Male)	.53	.08	< .001	.40	.70
Race (*ref*: White, not Hispanic)					
Black, not Hispanic	3.52	.77	< .001	2.29	5.43
All other races	1.39	.38	.230	.81	2.40
Rural (*ref*: Urban)	.86	.14	.347	.63	1.18
Work status (*ref*: Working for pay)					
Self-employed	2.25^a^	.63	.004	1.30	3.90
	1.37^b^	.37	.247	.80	2.34
	1.65^c^	.51	.106	.90	3.02
Retired	.32	.06	< .001	.22	.46
Disabled	.73	.20	.243	.43	1.24
Unemployed	1.15	.31	.605	.67	1.95
Others	.61	.17	.085	.35	1.07
**Human capital**					
Education (*ref*: High school or less)					
Associate’s degree	1.69	.30	.003	1.20	2.39
Bachelor’s degree	1.69	.34	.010	1.14	2.52
Master’s degree and above	1.50	.34	.072	.96	2.34
Health	1.14	.08	.047	1.00	1.30
Complete adult education/training	1.49	.22	.007	1.12	1.99
**Social capital**					
Married (*ref*: Not)	.81	.12	.156	.61	1.08
Volunteer (*ref*: Not)	1.78	.26	< .001	1.33	2.38
**Financial capital**					
Income	1.05	.06	.379	.94	1.18
Assets	1.00	.04	.957	.93	1.08
**Personal preferences and values**					
Meaning of work: Personal	1.19^a^	.05	< .001	1.10	1.29
	1.11^b^	.04	.001	1.05	1.18
	1.07^c^	.03	.005	1.02	1.12
Social	1.10	.02	< .001	1.05	1.14
Financial	1.08	.03	.005	1.02	1.13
Generativity	1.14	.04	< .001	1.07	1.21
Startup reason: (*ref*: Work for oneself)					
Make money	.44	.09	< .001	.29	.66
Meet social challenge, help others	.44^a^	.13	.005	.24	.78
	.64^b^	.16	.069	.40	1.03
	.64^c^	.17	.100	.38	1.09
Something else / Don’t know	.07	.02	< .001	.04	.12

*Note*. *uOR* = unadjusted odds ratio; *SE* = linearized standard error; *CI* = confidence interval. For the partial proportional odds models

^a^ odds ratio for very interested vs. less than very interested

^b^ odds ratio for somewhat or more interested vs not very much or less interested

^c^ odds ratio for not very much or more interested vs. not at all interested.

The bivariate results in Table 2 illustrate that interest in entrepreneurship was associated with several factors relating to demographics; human, social, and financial capital; and personal preferences and values. Specifically, those who were interested in entrepreneurship were younger (*uOR* = 0.90), less likely to identify as female (*uOR* = 0.53), and more likely to identify as black, compared to identifying as white (*uOR* = 3.52). Compared to those working for someone else, those who had the highest level of interest in entrepreneurship were more likely to be self-employed than those with less interest (*uOR* = 2.25). Compared to those working for pay for someone else, those who were interested in entrepreneurship were also less likely to be retired (*uOR* = 0.32). Interest in entrepreneurship showed a consistently significant association with human capital and personal preferences and values compared to social and financial capital. Regarding human capital and compared to having a high school education or less, those who had an associate’s degree (*uOR* = 1.69) or a bachelor’s degree (*uOR* = 1.69) had higher levels of interest in entrepreneurship. Interest was also associated with better health (*uOR* = 1.14) and having completed adult education or training (*uOR* = 1.49). Regarding social capital, volunteering was positively related to interest in entrepreneurship (*uOR* = 1.78). Regarding financial capital, income and assets were nonsignificant. For personal preferences and values, reporting higher levels in each of the four domains (personal, social, financial, and generativity) of the Meaning of Work Scale (*uOR* = 1.07–1.19) was positively related to interest in entrepreneurship. Further, and compared to the primary startup reason of working for oneself, those who stated that the primary reason would be to make money (*uOR* = 0.44) were less likely to report interest in entrepreneurship. Additionally, respondents with the highest level of interest in entrepreneurship were less likely to state that the primary reason for starting an organization would be to make money (*uOR* = .44) compared to those who said it would be to work for oneself.

The multivariable results of the partial proportional odds model are listed in Table 3. With a few exceptions, the multivariable results indicate similar, yet dampened, versions of the bivariate results. Consistent with the bivariate findings and while controlling for all other variables included in the analysis, the model shows that interest in entrepreneurship was negatively associated with age (*aOR* = 0.92) and identifying as female (*aOR* = 0.62). Additionally, identifying as black (*aOR* = 2.20) remained positively associated with interest in entrepreneurship. The odds ratios for these three variable had smaller magnitudes in the multivariable model. Being retired became nonsignificant. Finally, those with the highest interest in entrepreneurship, compared to those with somewhat or less interest, were more likely to report being self-employed (*aOR* = 3.45). Counter to the dampened odds ratios for several of the significant factors in the multivariable model, the magnitude of the odds ratio for the highest level of interest in entrepreneurship increased in the multivariable model.

**Table 3:** Multivariable partial proportional odds model for interest in entrepreneurship.

	aOR	SE	p	95% CI	
Demographics					
Age	0.92	0.02	< .001	0.88	0.95
Female (*ref*: Male)	0.62	0.10	.004	0.45	0.86
Race (*ref*: White, not Hispanic)					
Black, not Hispanic	2.20	0.61	.004	1.28	3.77
All other races	0.93	0.28	.807	0.51	1.68
Rural (*ref*: Urban)	0.97	0.18	.887	0.69	1.39
Work status (*ref*: Working for pay)					
Self-employed	3.45^a^	1.07	< .001	1.88	6.33
	1.80^b^	0.55	.055	0.99	3.30
	1.87^c^	0.76	.124	0.84	4.14
Retired	0.68	0.17	.138	0.41	1.13
Disabled	1.19	0.36	.577	0.65	2.17
Unemployed	1.08	0.38	.827	0.54	2.14
Others	0.81	0.23	.457	0.46	1.41
**Human capital**					
Education (*ref*: High school or less)					
Associate’s degree	1.31	0.29	.233	0.84	2.03
Bachelor’s degree	1.48	0.38	.127	0.89	2.45
Master’s degree and above	1.22	0.38	.529	0.66	2.26
Health	1.13	0.10	.156	0.95	1.34
Complete adult education/training	1.25	0.23	.234	0.87	1.80
**Social capital**					
Married (*ref*: Not)	0.76	0.14	.144	0.52	1.10
Volunteer (*ref*: Not)	1.61	0.27	.005	1.15	2.25
**Financial capital**					
Income	1.06	0.10	.560	0.88	1.27
Assets	0.98	0.06	.742	0.88	1.10
**Personal preferences and values**					
Meaning of work: Personal	1.11^a^	0.05	.023	1.01	1.22
	1.03^b^	0.04	.521	0.95	1.11
	0.97^c^	0.04	.419	0.90	1.04
Social	1.05	0.03	.065	1.00	1.11
Financial	0.95	0.03	.113	0.90	1.01
Generativity	1.07	0.05	.138	0.98	1.17
Startup reason: (*ref*: Work for oneself)					
Make money	0.56	0.13	.012	0.36	0.88
Meet social challenge, help others	0.37^a^	0.12	.002	0.20	0.70
	0.67^b^	0.17	.119	0.40	1.11
	0.73^c^	0.21	.283	0.41	1.30
Something else / Don’t know	0.09	0.03	< .001	0.05	0.16
Constant	0.87^a^	1.15	.915	0.06	11.73
	26.14^b^	32.83	.010	2.22	307.38
	201.69^c^	238.26	.000	19.85	2,048.85
*F test*: (32, 923) = 7.94, *p* < 0.001; *N* = 955					

*Note*. *aOR* = adjusted odds ratio; *SE* = linearized standard error; *CI* = confidence interval.

^a^ odds ratio for very interested vs. less than very interested

^b^ odds ratio for somewhat or more interested vs not very much or less interested

^c^ odds ratio for not very much or more interested vs. not at all interested.

While controlling for all other variables in the model, the human capital factors considered became nonsignificant. Considering social capital, those who volunteered reported a higher level of interest (*aOR* = 1.61). The two variables considered for financial capital—income and assets—remained nonsignificant.

Considering the relationship between personal preferences and values and interest in entrepreneurship, those with the highest interest in entrepreneurship, compared to those with somewhat or less interest, were more likely to report higher levels of the personal meaning of work (*aOR* = 1.11). However, the relationship between interest and the social, financial, and generative meanings of work became nonsignificant once controlling for all other variables in the model. Compared to those whose primary reason for starting an organization would be to work for themselves, those who reported the primary reason to make money (*aOR* = 0.56) were less likely to be interested in entrepreneurship. Additionally, respondents with the highest level of interest in entrepreneurship were less likely to state that the primary reason for starting an organization would be to meet a social challenge or help others (*uOR* = 0.37) compared to those who said it would be to work for oneself. Compared to those whose primary reason for starting an organization would be to work for themselves, those who reported the primary reason would be “something else” or “don’t know” were far less likely to be interested in entrepreneurship (*uOR* = 0.09).

### Sensitivity analysis

A series of sensitivity analyses were conducted to test our results using seemingly unrelated estimation. By design, our sample was heterogeneous in nature, including a cross-section of older Americans who were both in and outside of the workforce. However, it is possible that interest in entrepreneurship varied greatly due to one already being self-employed or retired, for example. As such, we used the *suest* program in Stata 15 to conduct a series of cumulative and binary seemingly unrelated estimation models to determine if the explanatory variables operated in similar ways for subsamples, allowing the parameters to vary at every level of the dependent variable. Samples compared included several workforce statuses (e.g., being self-employed or retired) as well as gender and age. While minor differences were found, they were infrequent and did not affect our overall conclusions. We therefore decided to keep our sample heterogeneous, working under the assumption that a person’s labor force status, for example, is separate from a person’s interest in entrepreneurship. Results from the mid-level (i.e., the dependent variable equals 1 for those reporting to be at least “somewhat” interested in entrepreneurship and 0 for those reporting to be “not too” or less interested) seemingly unrelated estimation sensitivity analyses are included in the supplemental files (S2–S7 Tables); results from the remaining two sets of analyses are similar. Further, we also include the results from the variance inflation factor tests (S1 Table) and a partial proportional odds model created using the iterative autofit feature in the *gologit2* user-written program (S8 Table), which allows all factors that failed the parallel lines assumption at the *p* < .05 level to vary in the supplemental files.

## Discussion

This study is the first to look at interest in entrepreneurship among older Americans using a multivariable framework. It finds that while there are differences in interest in starting a new business or nonprofit organizations in the next five to 10 years by key demographic variables, human and social capital, and personal preferences and values, there are also key areas where no difference in interest was established. These nonsignificant findings—urbanicity, those not currently in the labor force, educational factors and health, marital status, financial capital such as income and assets, and several personal preferences and values—point to the diversity and universal nature of interest in starting a new organization in later life. For example, while being married and having higher levels of income and assets are associated with later-life entrepreneurial activity [12], these factors were not found to be predictive of interest in entrepreneurship. This suggests that having a safety net—perhaps through higher or combined incomes, higher or shared assets, and spousal supports such as emotional encouragement, shared knowledge, and someone to help with family and household responsibilities—may buffer the relationship between interest and action. While these are individual- and family-level factors, similar arguments have been made for state- and national-level social insurance programs that reduce the consequences of entrepreneurial failure to increase entrepreneurship rates [73].

This study points to a large body of older Americans—more than an estimated 31 million—who report being at least somewhat interested in starting a new organization. Lenders; entrepreneurship workshop developers; management and social work schools, among others, that teach about and train potential business and social entrepreneurs; and policymakers should consider these results in relation to where they are seeking investment opportunities and attempting to spur economic growth. These results should cause one to question just who they envision an entrepreneur to be—and who they envision an entrepreneur *could* be—especially in light of the newest scholarship on the higher-than-expected age of the most successful entrepreneurs [6]. Indeed, older adults from a variety of backgrounds may be a smart—yet often overlooked—investment and economic growth opportunity.

These results also speak to the varied motivations for starting new organizations. Among those who reported the highest level of interest, for example, Table 1 shows that more than one in five (21%) stated that the primary reason would be to meet a social challenge or to help others, indicating a potentially large pool of older social—or “encore”—entrepreneurs [1]. Working for oneself was also found to be an important factor among those who reported the highest level of interest in all models, echoing previous research that has suggested the importance of autonomy in one’s work in later life, where it has been linked to the motivation to work, attitudes toward one’s job, and overall well-being [74]. Indeed, the relative importance of working for oneself as a motivator for becoming an entrepreneur was shown to grow with interest in our models.

Interest in entrepreneurship does decrease over time. These findings suggest that interest is at its peak at age 50 and declines slightly with every year. This is, after all, a time when people begin to think about retirement, and the notion of starting a completely new venture at this time may not be attractive. Further, many may fearful of losing access to important workplace benefits in later life that can be expensive without employer assistance, such as health insurance and retirement savings schemes. Indeed, older self-employed adults are less likely to be part of workplace pension and health insurance programs [13]. These barriers may be too expensive to overcome, as evidenced by the fact that rates of entrepreneurship increase after Americans turn 65, the general eligibility age for joining Medicare, the universal health insurance program for older Americans [75]. Indeed, scholars have called for increased levels of social protections—such as unemployment and “food stamp” (SNAP) benefits for entrepreneurs—to make it easier for potential entrepreneurs of all ages to begin their work [73].

In our final model, education was not found to be associated with interest in entrepreneurship. This is counter to two contradicting threads of scholarship regarding self-employment activity itself. First, among Americans aged 18 to 64, those with less than a high school education have rates of startup activity nearly twice that of those with a high school or more education, yet those with less education are also more likely to move into self-employment from unemployment [9]. In a major sense, they may have felt pushed into self-employment for lack of other employment options. Second, among Americans aged 51 and older, higher levels of education were associated with increased chances of becoming self-employed two years later from full-time wage-and-salary work among men [36] as well as from unemployment, disability, and retirement regardless of gender [10]. This suggests that education, especially for men, can bolster one’s ability to be pulled into entrepreneurship. Considering that much of the research conducted on interest in entrepreneurship has been fielded among those already enrolled in higher education institutions and particularly within business schools, much of our knowledge on this topic has been developed using samples that are much younger and more educated than the general population (e.g., [33,34,76]). In addition to considering older people, there is also a strong need to talk to those from more diverse socioeconomic backgrounds to assess their aspirations for entrepreneurial pursuits and how programs and policies can help them to be successful. These findings would also add a valuable voice to theory development.

This study found that older women are less likely to be interested in entrepreneurship than older men. This may be partially explained by labor force trends, as females are less likely to be working than males after the age of 55 [23]. As such, it is logical that they would also be less interested in starting their own organizations. As women’s labor force participation rates increase, this gap in interest may decrease. This difference may also be explained due to the wording of the question. As businesses led by older females are less likely to have employees than older males [13], it is possible that female respondents were less attracted to the terms “business” and “nonprofit organization” than males, as these terms may indicate the presence of employees. Previous scholarship has also documented the importance of perceptual differences by gender, with entrepreneurship being seen as a more masculine pursuit [33] and differences in nascent entrepreneurship by gender being attributed not to socioeconomic factors, but instead to perceptual factors, such as fear of failure and having sufficient skills to start a business [31].

This study also found that the older adults who identified as black were more likely than those who identified as white to be interested in entrepreneurship. Considering the findings in the final model, we found that the odds of older black, non-Hispanic Americans reporting the highest level of interest in entrepreneurship are more than twice that of older white, non-Hispanic Americans. Before controlling for potentially confounding variables, the results in the bivariate analysis indicate an even stronger relationship. This is counter to the consistent finding that black Americans of all ages are less likely to start businesses than white Americans [9]. Scholarship on where entrepreneurial opportunities currently exist for older Americans, if there are disparities in access to these opportunities by race and ethnicity, and how these disparities can be mitigated are important avenues to pursue. Indeed, Köllinger and Minniti [44] found that black Americans are twice as likely as white Americans to try starting a business, suggesting that differences in rates of entrepreneurship by race are not due to a lack of interest or effort, but instead due to structural and systemic barriers to entry that may lead to higher rates of failure.

These findings can be situated into the push/pull framework, which identifies older entrepreneurs as falling into two categories: those who *desire* to become self-employed for relatively positive reasons (i.e., they feel pulled into self-employment), and those who perceive the *need* to become self-employed for relatively negative or pragmatic reasons (i.e., they feel pushed into self-employment) (e.g., [2,5]). It is possible that this survey primarily identified respondents who felt pulled into becoming entrepreneurs in later life. However, many of those who may ultimately become entrepreneurs might feel pushed into this type of work, joining the ranks of what the Ewing Marion Kauffman Foundation has called “necessity” entrepreneurs [9]. These reasons could include needing additional income or lacking other job prospects. Further, these future “pushed” older entrepreneurs may not have been interested in entrepreneurship at the time of this survey and, perhaps a bit poignantly, may not even be interested in entrepreneurship once they move into this work. Future research that identifies supports for older adults who are pushed into entrepreneurship is warranted, as they likely have different needs than those who are pulled into this type of work.

### Limitations

A few limitations to this study should be noted. Although it was designed to be nationally representative, the survey used in this study did not oversample smaller racial and ethnic groups; therefore, only individuals who identified as non-Hispanic white and non-Hispanic black could be specified in the model, with all other individuals included in a third category that is difficult to interpret. Future scholarship that aims to understand the aspirations of members of non-majority groups should consider oversampling from these populations for surveys, in addition to in-depth qualitative research. This research is also cross-sectional, preventing an understanding of the pathway from interest to action among potential entrepreneurs in later life. Future longitudinal studies that test what factors lead to interest in entrepreneurship and in turn lead to entrepreneurial activity should be conducted. Finally, this study investigated individual-level factors and how they relate to interest in entrepreneurship; however, factors related to communities, society, and the broader economy are likely to have an influence as well [2].

### Implications

This study highlights that interest in entrepreneurship in later life is not the sole province of those with higher levels of human, social, and financial capital, nor can it be explained by many personal preferences and values. Among adults aged 50 to 70, there is a sizable level of interest in starting new businesses and nonprofit organizations, and those interested are diverse in many ways. Those seeking to support nascent entrepreneurs should consider older individuals as potential investment opportunities, and those seeking to encourage later-life entrepreneurship should look to new communities for potential start-up creation. This study complements previous scholarship that theorized and documented gaps between entrepreneurial interest and action by race, gender, and other variables (e.g., [2,31,44]), illustrating that these gaps may be present among older adults. While not possible for this study, future research should attempt to identify where gaps exist between entrepreneurial interest and action.

Scholarship on this topic should not end here. Among adults past midlife, future research should investigate the causes of interest in entrepreneurship, as well as the programs and policies that can support a diverse group of older adults in starting and managing successful ventures. Further, scholarship is also needed on how later-life entrepreneurship influences individuals’ mental, physical, and financial well-being; if entrepreneurs’ levels of human, social, and financial capital moderate these outcomes; and what work-related characteristics, such as industry, incorporation status, and work schedules influence these outcomes. Finally, a new line of research that examines attributes of existing entrepreneurship training programs that are designed for or inclusive of people past midlife is needed. In a rapidly aging society, it is important that we continue to investigate how to increase successful outcomes for a diverse range of older adults.

## Supporting information

S1 Table Variance inflation factor results. (DOCX)

S2 Table Logistic regression on interest in entrepreneurship, by gender. (DOCX)

S3 Table Logistic regression on interest in entrepreneurship, by work status. (DOCX)

S4 Table Logistic regression on interest in entrepreneurship, by retirement status. (DOCX)

S5 Table Logistic regression on interest in entrepreneurship, by self-employment status. (DOCX)

S6 Table Logistic regression on interest in entrepreneurship, by age. (DOCX)

S7 Table Logistic regression on interest in entrepreneurship, by “something else/don't know” startup purpose. (DOCX)

S8 Table Multivariable partial proportional odds model using the autofit feature in *Gologit2*. (DOCX)

S1 Dataset 2014 encore career survey. All data used in this study’s analysis are included as supporting information in a Stata data file.
(DTA)
