# Supplementary material for: The diversity of interest in later-life entrepreneurship: Results from a nationally representative survey of Americans aged 50 to 70
Source: PLoS One. 2019 Jun 5;14(6):e0217971. doi: 10.1371/journal.pone.0217971 (PMC6550427; doi:10.1371/journal.pone.0217971)
Supplement: S1 Table — (DOCX) [file pone.0217971.s001.docx]

**S1 Table. Variance Inflation Factor Results**

|  | **VIF** |  | **1/VIF** |
| --- | --- | --- | --- |
| **Demographics** |  |  |  |
| Age | 1.47 |  | 0.678913 |
| Gender | 1.14 |  | 0.880525 |
| Race (*ref*: White, not Hispanic) |  |  |  |
| Black, not Hispanic | 1.08 |  | 0.925640 |
| All other races | 1.08 |  | 0.929988 |
| Rural (*ref*: Urban) | 1.08 |  | 0.924346 |
| Work status (*ref*: Working for pay) |  |  |  |
| Self-employed | 1.25 |  | 0.799331 |
| Retired | 1.92 |  | 0.521393 |
| Disabled | 1.59 |  | 0.627867 |
| Unemployed | 1.21 |  | 0.829248 |
| Others | 1.26 |  | 0.790700 |
| **Human capital** |  |  |  |
| Education (*ref*: High school or less) |  |  |  |
| Associate’s degree | 1.90 |  | 0.525928 |
| Bachelor’s degree | 1.96 |  | 0.511093 |
| Master’s degree and above | 1.73 |  | 0.578602 |
| Health | 1.28 |  | 0.779779 |
| Complete adult education/training | 1.15 |  | 0.867162 |
| **Social capital** |  |  |  |
| Married (*ref*: Not) | 1.33 |  | 0.751651 |
| Volunteer (*ref*: Not) | 1.17 |  | 0.851457 |
| **Financial capital** |  |  |  |
| Income | 2.04 |  | 0.489796 |
| Assets | 1.78 |  | 0.563296 |
| **Personal preferences and values** |  |  |  |
| Startup reason: (*ref:* Work for oneself) |  |  |  |
| Make money | 2.02 |  | 0.494231 |
| Meet social challenge, help others | 1.85 |  | 0.541981 |
| Something else/Don’t know | 1.87 |  | 0.536095 |
| Meaning of work: Personal | 2.35 |  | 0.424868 |
| Social | 1.85 |  | 0.539806 |
| Financial | 1.47 |  | 0.680872 |
| Generativity | 2.08 |  | 0.480238 |
| Mean VIF | 1.57 |  |  |

*Note*. VIF = variance inflation factor. Results obtained after running an OLS regression on interest in entrepreneurship.
