# Supplementary material for: The diversity of interest in later-life entrepreneurship: Results from a nationally representative survey of Americans aged 50 to 70
Source: PLoS One. 2019 Jun 5;14(6):e0217971. doi: 10.1371/journal.pone.0217971 (PMC6550427; doi:10.1371/journal.pone.0217971)
Supplement: S2 Table — (DOCX) [file pone.0217971.s002.docx]

**S2 Table. Logistic Regression on Interest in Entrepreneurship, by Gender**

|  |  |  | **Women** | | | |  | **Men** | | | |
| --- | --- | --- | --- | --- | --- | --- | --- | --- | --- | --- | --- |
|  | **SUE** |  | **aOR** | **SE** | **p** | **95% CI** |  | **aOR** | **SE** | **p** | **95% CI** |
| **Demographics** |  |  |  |  |  |  |  |  |  |  |  |
| Age |  |  | 0.93 | 0.03 | 0.011 | 0.88 - 0.98 |  | 0.90 | 0.02 | 0.000 | 0.85 - 0.94 |
| Gender |  |  | - | - | - | - |  | - | - | - | - |
| Race (*ref*: White, not Hispanic) |  |  |  |  |  |  |  |  |  |  |  |
| Black, not Hispanic |  |  | 4.05 | 2.15 | 0.009 | 1.42 - 11.50 |  | 1.88 | 0.91 | 0.195 | 0.72 - 4.86 |
| All other races | * |  | 0.34 | 0.18 | 0.042 | 0.12 - 0.96 |  | 3.10 | 1.42 | 0.014 | 1.26 - 7.62 |
| Rural (*ref*: Urban) |  |  | 1.73 | 0.50 | 0.056 | 0.98 - 3.04 |  | 1.02 | 0.29 | 0.957 | 0.58 - 1.76 |
| Work status (*ref*: Working for pay) |  |  |  |  |  |  |  |  |  |  |  |
| Self-employed |  |  | 2.29 | 1.43 | 0.186 | 0.67 - 7.81 |  | 0.98 | 0.41 | 0.958 | 0.43 - 2.21 |
| Retired |  |  | 0.55 | 0.22 | 0.126 | 0.25 - 1.19 |  | 0.67 | 0.27 | 0.320 | 0.30 - 1.48 |
| Disabled | * |  | 0.39 | 0.19 | 0.050 | 0.15 - 1.00 |  | 1.71 | 0.81 | 0.257 | 0.68 - 4.33 |
| Unemployed |  |  | 0.89 | 0.53 | 0.848 | 0.28 - 2.84 |  | 0.68 | 0.38 | 0.493 | 0.22 - 2.06 |
| Others |  |  | 0.80 | 0.35 | 0.613 | 0.35 - 1.87 |  | 1.73 | 1.20 | 0.426 | 0.45 - 6.71 |
| **Human capital** |  |  |  |  |  |  |  |  |  |  |  |
| Education (*ref*: High school or less) |  |  |  |  |  |  |  |  |  |  |  |
| Associate’s degree |  |  | 1.00 | 0.34 | 0.997 | 0.51 - 1.95 |  | 1.26 | 0.43 | 0.502 | 0.64 - 2.45 |
| Bachelor’s degree |  |  | 1.76 | 0.78 | 0.205 | 0.74 - 4.20 |  | 1.40 | 0.57 | 0.418 | 0.62 - 3.13 |
| Master’s degree and above |  |  | 1.77 | 0.91 | 0.265 | 0.65 - 4.84 |  | 0.80 | 0.39 | 0.651 | 0.31 - 2.07 |
| Health |  |  | 0.91 | 0.13 | 0.533 | 0.69 - 1.22 |  | 1.36 | 0.20 | 0.035 | 1.02 - 1.80 |
| Complete adult education/training |  |  | 1.88 | 0.57 | 0.039 | 1.03 - 3.41 |  | 0.93 | 0.28 | 0.813 | 0.52 - 1.67 |
| **Social capital** |  |  |  |  |  |  |  |  |  |  |  |
| Married (*ref*: Not) | * |  | 0.54 | 0.17 | 0.055 | 0.29 - 1.01 |  | 1.30 | 0.40 | 0.395 | 0.71 - 2.38 |
| Volunteer (*ref*: Not) |  |  | 1.62 | 0.47 | 0.092 | 0.92 - 2.86 |  | 1.57 | 0.44 | 0.105 | 0.91 - 2.71 |
| **Financial capital** |  |  |  |  |  |  |  |  |  |  |  |
| Income |  |  | 1.03 | 0.16 | 0.839 | 0.76 - 1.41 |  | 1.14 | 0.18 | 0.378 | 0.85 - 1.55 |
| Assets |  |  | 0.80 | 0.08 | 0.025 | 0.66 - 0.97 |  | 0.98 | 0.10 | 0.848 | 0.80 - 1.20 |
| **Personal preferences and values** |  |  |  |  |  |  |  |  |  |  |  |
| Startup reason: (*ref:* Work for oneself) |  |  |  |  |  |  |  |  |  |  |  |
| Make money |  |  | 0.63 | 0.23 | 0.200 | 0.31 - 1.28 |  | 0.48 | 0.18 | 0.049 | 0.24 - 1.00 |
| Meet social challenge, help others |  |  | 0.38 | 0.16 | 0.020 | 0.17 - 0.86 |  | 0.78 | 0.32 | 0.533 | 0.35 - 1.72 |
| Something else/Don’t know |  |  | 0.08 | 0.04 | 0.000 | 0.03 - 0.22 |  | 0.02 | 0.02 | 0.000 | 0.01 - 0.09 |
| Meaning of work: Personal |  |  | 1.05 | 0.06 | 0.371 | 0.94 - 1.17 |  | 0.99 | 0.05 | 0.915 | 0.90 - 1.10 |
| Social |  |  | 1.01 | 0.04 | 0.753 | 0.93 - 1.10 |  | 1.08 | 0.04 | 0.052 | 1.00 - 1.17 |
| Financial |  |  | 0.94 | 0.05 | 0.206 | 0.85 - 1.04 |  | 0.95 | 0.05 | 0.359 | 0.86 - 1.06 |
| Generativity |  |  | 1.05 | 0.07 | 0.466 | 0.92 - 1.20 |  | 1.09 | 0.07 | 0.135 | 0.97 - 1.23 |
| Constant |  |  | 41.98 | 79.86 | 0.050 | 1.00 - 1,755.07 |  | 32.31 | 57.48 | 0.051 | 0.98 - 1,060.84 |

*Note*. The binary dependent variable included “very interested” or “somewhat interested” = 1 and “not too interested” and “not at all interested” = 0; *SUE* = seemingly unrelated estimation results, indicating differences between the parameters of both groups with *p* < .05 indicated by *; *aOR* = adjusted odds ratio; *SE* = linearized standard error; *CI* = confidence interval.
