# Supplementary material for: The diversity of interest in later-life entrepreneurship: Results from a nationally representative survey of Americans aged 50 to 70
Source: PLoS One. 2019 Jun 5;14(6):e0217971. doi: 10.1371/journal.pone.0217971 (PMC6550427; doi:10.1371/journal.pone.0217971)
Supplement: S3 Table — (DOCX) [file pone.0217971.s003.docx]

**S3 Table. Logistic Regression on Interest in Entrepreneurship, by Work Status**

|  |  |  | **Not Working** | | | |  | **Working** | | | |
| --- | --- | --- | --- | --- | --- | --- | --- | --- | --- | --- | --- |
|  | **SUE** |  | **aOR** | **SE** | **p** | **95% CI** |  | **aOR** | **SE** | **p** | **95% CI** |
| **Demographics** |  |  |  |  |  |  |  |  |  |  |  |
| Age |  |  | 0.88 | 0.02 | 0.000 | 0.84 - 0.92 |  | 0.94 | 0.03 | 0.020 | 0.89 - 0.99 |
| Gender |  |  | 0.61 | 0.18 | 0.086 | 0.34 - 1.07 |  | 0.64 | 0.17 | 0.101 | 0.37 - 1.09 |
| Race (*ref*: White, not Hispanic) |  |  |  |  |  |  |  |  |  |  |  |
| Black, not Hispanic |  |  | 2.39 | 1.35 | 0.126 | 0.78 - 7.27 |  | 1.98 | 0.85 | 0.113 | 0.85 - 4.59 |
| All other races |  |  | 1.09 | 0.53 | 0.856 | 0.42 - 2.81 |  | 1.24 | 0.53 | 0.622 | 0.53 - 2.89 |
| Rural (*ref*: Urban) |  |  | 1.41 | 0.44 | 0.269 | 0.76 - 2.62 |  | 0.99 | 0.28 | 0.974 | 0.57 - 1.71 |
| Work status (*ref*: Working for pay) |  |  |  |  |  |  |  |  |  |  |  |
| Self-employed |  |  | - | - | - | - |  | - | - | - | - |
| Retired |  |  | - | - | - | - |  | - | - | - | - |
| Disabled |  |  | - | - | - | - |  | - | - | - | - |
| Unemployed |  |  | - | - | - | - |  | - | - | - | - |
| Others |  |  | - | - | - | - |  | - | - | - | - |
| **Human capital** |  |  |  |  |  |  |  |  |  |  |  |
| Education (*ref*: High school or less) |  |  |  |  |  |  |  |  |  |  |  |
| Associate’s degree |  |  | 0.75 | 0.26 | 0.396 | 0.38 - 1.47 |  | 1.61 | 0.60 | 0.198 | 0.78 - 3.34 |
| Bachelor’s degree |  |  | 1.94 | 0.78 | 0.098 | 0.88 - 4.26 |  | 1.07 | 0.45 | 0.874 | 0.47 - 2.45 |
| Master’s degree and above |  |  | 0.98 | 0.53 | 0.968 | 0.34 - 2.85 |  | 1.22 | 0.61 | 0.697 | 0.45 - 3.27 |
| Health |  |  | 1.00 | 0.14 | 0.994 | 0.76 - 1.31 |  | 1.39 | 0.21 | 0.029 | 1.03 - 1.88 |
| Complete adult education/training |  |  | 1.67 | 0.51 | 0.095 | 0.91 - 3.05 |  | 1.15 | 0.35 | 0.650 | 0.63 - 2.09 |
| **Social capital** |  |  |  |  |  |  |  |  |  |  |  |
| Married (*ref*: Not) |  |  | 0.80 | 0.26 | 0.497 | 0.42 - 1.53 |  | 0.91 | 0.27 | 0.748 | 0.50 - 1.64 |
| Volunteer (*ref*: Not) |  |  | 1.52 | 0.50 | 0.208 | 0.79 - 2.91 |  | 1.87 | 0.53 | 0.028 | 1.07 - 3.27 |
| **Financial capital** |  |  |  |  |  |  |  |  |  |  |  |
| Income |  |  | 1.35 | 0.24 | 0.094 | 0.95 - 1.90 |  | 0.90 | 0.14 | 0.492 | 0.67 - 1.21 |
| Assets |  |  | 0.80 | 0.09 | 0.037 | 0.65 - 0.99 |  | 1.02 | 0.10 | 0.844 | 0.84 - 1.24 |
| **Personal preferences and values** |  |  |  |  |  |  |  |  |  |  |  |
| Startup reason: (*ref:* Work for oneself) |  |  |  |  |  |  |  |  |  |  |  |
| Make money |  |  | 0.64 | 0.26 | 0.280 | 0.29 - 1.44 |  | 0.38 | 0.13 | 0.004 | 0.20 - 0.74 |
| Meet social challenge, help others |  |  | 0.58 | 0.27 | 0.249 | 0.23 - 1.46 |  | 0.75 | 0.29 | 0.460 | 0.35 - 1.62 |
| Something else/Don’t know |  |  | 0.05 | 0.03 | 0.000 | 0.01 - 0.19 |  | 0.05 | 0.03 | 0.000 | 0.02 - 0.15 |
| Meaning of work: Personal |  |  | 1.07 | 0.07 | 0.258 | 0.95 - 1.21 |  | 0.99 | 0.05 | 0.910 | 0.90 - 1.09 |
| Social |  |  | 1.06 | 0.05 | 0.225 | 0.97 - 1.15 |  | 1.02 | 0.04 | 0.543 | 0.95 - 1.10 |
| Financial | * |  | 0.86 | 0.05 | 0.011 | 0.77 - 0.97 |  | 1.02 | 0.05 | 0.618 | 0.93 - 1.13 |
| Generativity |  |  | 1.02 | 0.08 | 0.823 | 0.88 - 1.18 |  | 1.06 | 0.06 | 0.285 | 0.95 - 1.19 |
| Constant |  |  | 575.56 | 989.10 | 0.000 | 19.75 - 16,774.63 |  | 7.79 | 13.53 | 0.238 | 0.26 - 235.65 |

*Note*. The binary dependent variable included “very interested” or “somewhat interested” = 1 and “not too interested” and “not at all interested” = 0; “Not working” includes those who report being retired, disabled, unemployed, or in another category, and “Working” includes those who report working for pay or being self-employed; *SUE* = seemingly unrelated estimation results, indicating differences between the parameters of both groups with *p* < .05 indicated by *; *aOR* = adjusted odds ratio; *SE* = linearized standard error; *CI* = confidence interval.
