# Supplementary material for: The diversity of interest in later-life entrepreneurship: Results from a nationally representative survey of Americans aged 50 to 70
Source: PLoS One. 2019 Jun 5;14(6):e0217971. doi: 10.1371/journal.pone.0217971 (PMC6550427; doi:10.1371/journal.pone.0217971)
Supplement: S5 Table — (DOCX) [file pone.0217971.s005.docx]

**S5 Table. Logistic Regression on Interest in Entrepreneurship, by Self-Employment Status**

|  |  |  | **Self-Employed** | | | |  | **Not Self-Employed** | | | |
| --- | --- | --- | --- | --- | --- | --- | --- | --- | --- | --- | --- |
|  | **SUE** |  | **aOR** | **SE** | **p** | **95% CI** |  | **aOR** | **SE** | **p** | **95% CI** |
| **Demographics** |  |  |  |  |  |  |  |  |  |  |  |
| Age |  |  | 0.94 | 0.06 | 0.354 | 0.83 - 1.07 |  | 0.90 | 0.02 | 0.000 | 0.87 - 0.93 |
| Gender |  |  | 1.50 | 0.90 | 0.494 | 0.47 - 4.84 |  | 0.51 | 0.10 | 0.001 | 0.34 - 0.75 |
| Race (*ref*: White, not Hispanic) |  |  |  |  |  |  |  |  |  |  |  |
| Black, not Hispanic |  |  | 0.92 | 0.93 | 0.933 | 0.13 - 6.63 |  | 2.95 | 1.13 | 0.005 | 1.39 - 6.26 |
| All other races |  |  | 0.57 | 0.47 | 0.494 | 0.11 - 2.89 |  | 1.26 | 0.42 | 0.494 | 0.65 - 2.44 |
| Rural (*ref*: Urban) |  |  | 1.40 | 0.91 | 0.607 | 0.39 - 5.03 |  | 1.13 | 0.25 | 0.586 | 0.73 - 1.74 |
| Work status (*ref*: Working for pay) |  |  |  |  |  |  |  |  |  |  |  |
| Self-employed |  |  | - | - | - | - |  | - | - | - | - |
| Retired |  |  | - | - | - | - |  | - | - | - | - |
| Disabled |  |  | - | - | - | - |  | - | - | - | - |
| Unemployed |  |  | - | - | - | - |  | - | - | - | - |
| Others |  |  | - | - | - | - |  | - | - | - | - |
| **Human capital** |  |  |  |  |  |  |  |  |  |  |  |
| Education (*ref*: High school or less) |  |  |  |  |  |  |  |  |  |  |  |
| Associate’s degree | * |  | 7.64 | 7.43 | 0.037 | 1.13 - 51.55 |  | 0.97 | 0.25 | 0.897 | 0.58 - 1.61 |
| Bachelor’s degree |  |  | 3.00 | 2.85 | 0.247 | 0.47 - 19.32 |  | 1.69 | 0.50 | 0.075 | 0.95 - 3.01 |
| Master’s degree and above |  |  | 2.06 | 2.30 | 0.519 | 0.23 - 18.39 |  | 1.50 | 0.58 | 0.291 | 0.71 - 3.20 |
| Health |  |  | 1.09 | 0.39 | 0.806 | 0.55 - 2.18 |  | 1.09 | 0.11 | 0.420 | 0.89 - 1.34 |
| Complete adult education/training |  |  | 0.59 | 0.45 | 0.486 | 0.13 - 2.63 |  | 1.25 | 0.28 | 0.318 | 0.81 - 1.94 |
| **Social capital** |  |  |  |  |  |  |  |  |  |  |  |
| Married (*ref*: Not) |  |  | 1.15 | 0.68 | 0.812 | 0.36 - 3.69 |  | 0.83 | 0.19 | 0.424 | 0.53 - 1.30 |
| Volunteer (*ref*: Not) |  |  | 1.67 | 1.07 | 0.422 | 0.48 - 5.84 |  | 1.71 | 0.39 | 0.017 | 1.10 - 2.67 |
| **Financial capital** |  |  |  |  |  |  |  |  |  |  |  |
| Income |  |  | 1.38 | 0.41 | 0.281 | 0.77 - 2.49 |  | 1.04 | 0.12 | 0.741 | 0.83 - 1.30 |
| Assets |  |  | 0.89 | 0.19 | 0.577 | 0.59 - 1.35 |  | 0.91 | 0.07 | 0.198 | 0.79 - 1.05 |
| **Personal preferences and values** |  |  |  |  |  |  |  |  |  |  |  |
| Startup reason: (*ref:* Work for oneself) |  |  |  |  |  |  |  |  |  |  |  |
| Make money |  |  | 0.81 | 0.48 | 0.722 | 0.25 - 2.62 |  | 0.44 | 0.12 | 0.003 | 0.25 - 0.76 |
| Meet social challenge, help others |  |  | 2.32 | 2.03 | 0.336 | 0.42 - 12.92 |  | 0.48 | 0.15 | 0.020 | 0.26 - 0.89 |
| Something else/Don’t know |  |  | 0.23 | 0.26 | 0.186 | 0.03 - 2.03 |  | 0.04 | 0.02 | 0.000 | 0.02 - 0.10 |
| Meaning of work: Personal |  |  | 1.04 | 0.10 | 0.700 | 0.86 - 1.24 |  | 1.03 | 0.05 | 0.484 | 0.95 - 1.13 |
| Social |  |  | 0.95 | 0.08 | 0.535 | 0.81 - 1.12 |  | 1.05 | 0.03 | 0.124 | 0.99 - 1.11 |
| Financial | * |  | 1.17 | 0.11 | 0.102 | 0.97 - 1.40 |  | 0.93 | 0.04 | 0.078 | 0.86 - 1.01 |
| Generativity |  |  | 0.95 | 0.10 | 0.625 | 0.78 - 1.16 |  | 1.06 | 0.06 | 0.276 | 0.95 - 1.19 |
| Constant |  |  | 2.60 | 9.30 | 0.790 | 0.00 - 2,931.85 |  | 156.39 | 219.15 | 0.000 | 10.00 - 2,445.68 |

*Note*. The binary dependent variable included “very interested” or “somewhat interested” = 1 and “not too interested” and “not at all interested” = 0; “Self-Employed” includes those who report being self-employed, and “Not Self-Employed” includes all others; *SUE* = seemingly unrelated estimation results, indicating differences between the parameters of both groups with *p* < .05 indicated by *; *aOR* = adjusted odds ratio; *SE* = linearized standard error; *CI* = confidence interval.
