# Supplementary material for: The diversity of interest in later-life entrepreneurship: Results from a nationally representative survey of Americans aged 50 to 70
Source: PLoS One. 2019 Jun 5;14(6):e0217971. doi: 10.1371/journal.pone.0217971 (PMC6550427; doi:10.1371/journal.pone.0217971)
Supplement: S6 Table — (DOCX) [file pone.0217971.s006.docx]

**S6 Table. Logistic Regression on Interest in Entrepreneurship, by Age**

|  |  |  | **<62 years old** | | | |  | **62+ years old** | | | |
| --- | --- | --- | --- | --- | --- | --- | --- | --- | --- | --- | --- |
|  | **SUE** |  | **aOR** | **SE** | **p** | **95% CI** |  | **aOR** | **SE** | **p** | **95% CI** |
| **Demographics** |  |  |  |  |  |  |  |  |  |  |  |
| Age |  |  | - | - | - | - |  | - | - | - | - |
| Gender |  |  | 0.53 | 0.12 | 0.005 | 0.34 - 0.82 |  | 0.71 | 0.28 | 0.380 | 0.33 - 1.53 |
| Race (*ref*: White, not Hispanic) |  |  |  |  |  |  |  |  |  |  |  |
| Black, not Hispanic |  |  | 2.56 | 1.04 | 0.021 | 1.15 - 5.68 |  | 2.26 | 1.49 | 0.216 | 0.62 - 8.22 |
| All other races |  |  | 1.31 | 0.46 | 0.436 | 0.66 - 2.60 |  | 0.30 | 0.28 | 0.203 | 0.05 - 1.93 |
| Rural (*ref*: Urban) |  |  | 1.21 | 0.29 | 0.424 | 0.76 - 1.93 |  | 1.05 | 0.44 | 0.909 | 0.46 - 2.40 |
| Work status (*ref*: Working for pay) |  |  |  |  |  |  |  |  |  |  |  |
| Self-employed |  |  | 1.27 | 0.46 | 0.514 | 0.62 - 2.57 |  | 1.55 | 1.40 | 0.629 | 0.26 - 9.16 |
| Retired |  |  | 0.86 | 0.32 | 0.681 | 0.41 - 1.78 |  | 0.36 | 0.18 | 0.043 | 0.14 - 0.97 |
| Disabled |  |  | 0.90 | 0.32 | 0.762 | 0.44 - 1.81 |  | 0.13 | 0.14 | 0.060 | 0.02 - 1.09 |
| Unemployed |  |  | 0.72 | 0.31 | 0.452 | 0.30 - 1.70 |  | 2.61 | 2.45 | 0.305 | 0.42 - 16.43 |
| Others |  |  | 0.92 | 0.36 | 0.835 | 0.43 - 1.99 |  | 0.19 | 0.18 | 0.086 | 0.03 - 1.27 |
| **Human capital** |  |  |  |  |  |  |  |  |  |  |  |
| Education (*ref*: High school or less) |  |  |  |  |  |  |  |  |  |  |  |
| Associate’s degree |  |  | 1.20 | 0.34 | 0.517 | 0.69 - 2.08 |  | 1.52 | 0.82 | 0.432 | 0.53 - 4.37 |
| Bachelor’s degree |  |  | 1.55 | 0.51 | 0.186 | 0.81 - 2.97 |  | 2.50 | 1.49 | 0.125 | 0.78 - 8.06 |
| Master’s degree and above |  |  | 1.06 | 0.46 | 0.900 | 0.45 - 2.50 |  | 2.75 | 1.89 | 0.141 | 0.71 - 10.59 |
| Health |  |  | 1.13 | 0.14 | 0.325 | 0.89 - 1.44 |  | 1.17 | 0.24 | 0.432 | 0.79 - 1.76 |
| Complete adult education/training |  |  | 1.44 | 0.37 | 0.155 | 0.87 - 2.38 |  | 0.84 | 0.32 | 0.641 | 0.40 - 1.77 |
| **Social capital** |  |  |  |  |  |  |  |  |  |  |  |
| Married (*ref*: Not) |  |  | 0.81 | 0.20 | 0.386 | 0.50 - 1.30 |  | 0.66 | 0.30 | 0.362 | 0.27 - 1.61 |
| Volunteer (*ref*: Not) | * |  | 2.33 | 0.55 | 0.000 | 1.47 - 3.70 |  | 0.45 | 0.19 | 0.065 | 0.19 - 1.05 |
| **Financial capital** |  |  |  |  |  |  |  |  |  |  |  |
| Income |  |  | 0.97 | 0.12 | 0.823 | 0.76 - 1.24 |  | 1.46 | 0.33 | 0.097 | 0.93 - 2.27 |
| Assets |  |  | 0.99 | 0.08 | 0.882 | 0.84 - 1.16 |  | 0.74 | 0.10 | 0.027 | 0.57 - 0.97 |
| **Personal preferences and values** |  |  |  |  |  |  |  |  |  |  |  |
| Startup reason: (*ref:* Work for oneself) |  |  |  |  |  |  |  |  |  |  |  |
| Make money |  |  | 0.57 | 0.17 | 0.052 | 0.32 - 1.01 |  | 0.25 | 0.13 | 0.007 | 0.09 - 0.68 |
| Meet social challenge, help others |  |  | 0.58 | 0.20 | 0.121 | 0.29 - 1.16 |  | 0.38 | 0.21 | 0.085 | 0.13 - 1.14 |
| Something else/Don’t know |  |  | 0.06 | 0.03 | 0.000 | 0.03 - 0.15 |  | 0.02 | 0.02 | 0.000 | 0.00 - 0.12 |
| Meaning of work: Personal |  |  | 1.05 | 0.05 | 0.301 | 0.96 - 1.14 |  | 1.00 | 0.07 | 0.992 | 0.86 - 1.16 |
| Social |  |  | 1.04 | 0.04 | 0.301 | 0.97 - 1.11 |  | 1.07 | 0.07 | 0.312 | 0.94 - 1.21 |
| Financial |  |  | 0.94 | 0.04 | 0.185 | 0.87 - 1.03 |  | 0.91 | 0.07 | 0.193 | 0.78 - 1.05 |
| Generativity |  |  | 1.01 | 0.06 | 0.859 | 0.90 - 1.13 |  | 1.17 | 0.09 | 0.053 | 1.00 - 1.37 |
| Constant |  |  | 0.38 | 0.34 | 0.278 | 0.06 - 2.21 |  | 0.22 | 0.29 | 0.259 | 0.02 - 3.08 |

*Note*. The binary dependent variable included “very interested” or “somewhat interested” = 1 and “not too interested” and “not at all interested” = 0; *SUE* = seemingly unrelated estimation results, indicating differences between the parameters of both groups with *p* < .05 indicated by *; *aOR* = adjusted odds ratio; *SE* = linearized standard error; *CI* = confidence interval.
