# Supplementary material for: The diversity of interest in later-life entrepreneurship: Results from a nationally representative survey of Americans aged 50 to 70
Source: PLoS One. 2019 Jun 5;14(6):e0217971. doi: 10.1371/journal.pone.0217971 (PMC6550427; doi:10.1371/journal.pone.0217971)
Supplement: S7 Table — (DOCX) [file pone.0217971.s007.docx]

**S7 Table. Logistic Regression on Interest in Entrepreneurship, by “Something Else/Don't Know” Startup Purpose**

|  |  |  | **All respondents** | | | |  | **Without “something else/don’t know”** | | | |
| --- | --- | --- | --- | --- | --- | --- | --- | --- | --- | --- | --- |
|  | **SUE** |  | **aOR** | **SE** | **p** | **95% CI** |  | **aOR** | **SE** | **p** | **95% CI** |
| **Demographics** |  |  |  |  |  |  |  |  |  |  |  |
| Age |  |  | 0.91 | 0.02 | 0.000 | 0.88 - 0.95 |  | 0.91 | 0.02 | 0.000 | 0.88 - 0.95 |
| Gender |  |  | 0.60 | 0.11 | 0.006 | 0.41 - 0.86 |  | 0.54 | 0.11 | 0.002 | 0.37 - 0.80 |
| Race (*ref*: White, not Hispanic) |  |  |  |  |  |  |  |  |  |  |  |
| Black, not Hispanic |  |  | 2.70 | 0.95 | 0.005 | 1.35 - 5.40 |  | 2.44 | 0.85 | 0.010 | 1.24 - 4.83 |
| All other races |  |  | 1.18 | 0.37 | 0.599 | 0.64 - 2.19 |  | 1.19 | 0.40 | 0.598 | 0.62 - 2.30 |
| Rural (*ref*: Urban) |  |  | 1.22 | 0.24 | 0.328 | 0.82 - 1.81 |  | 1.12 | 0.23 | 0.593 | 0.74 - 1.67 |
| Work status (*ref*: Working for pay) |  |  |  |  |  |  |  |  |  |  |  |
| Self-employed |  |  | 1.42 | 0.47 | 0.298 | 0.73 - 2.73 |  | 1.30 | 0.44 | 0.435 | 0.67 - 2.51 |
| Retired |  |  | 0.70 | 0.20 | 0.217 | 0.40 - 1.23 |  | 0.72 | 0.21 | 0.258 | 0.40 - 1.28 |
| Disabled |  |  | 0.88 | 0.30 | 0.712 | 0.46 - 1.71 |  | 0.89 | 0.32 | 0.752 | 0.45 - 1.79 |
| Unemployed |  |  | 0.82 | 0.34 | 0.628 | 0.36 - 1.86 |  | 0.78 | 0.33 | 0.555 | 0.34 - 1.79 |
| Others |  |  | 0.82 | 0.29 | 0.568 | 0.41 - 1.63 |  | 0.89 | 0.32 | 0.746 | 0.43 - 1.82 |
| **Human capital** |  |  |  |  |  |  |  |  |  |  |  |
| Education (*ref*: High school or less) |  |  |  |  |  |  |  |  |  |  |  |
| Associate’s degree |  |  | 1.19 | 0.29 | 0.480 | 0.74 - 1.91 |  | 1.24 | 0.31 | 0.385 | 0.76 - 2.03 |
| Bachelor’s degree |  |  | 1.67 | 0.47 | 0.072 | 0.96 - 2.91 |  | 1.50 | 0.43 | 0.157 | 0.85 - 2.65 |
| Master’s degree and above |  |  | 1.41 | 0.51 | 0.343 | 0.69 - 2.86 |  | 1.38 | 0.51 | 0.378 | 0.67 - 2.85 |
| Health |  |  | 1.11 | 0.11 | 0.325 | 0.91 - 1.35 |  | 1.11 | 0.12 | 0.309 | 0.91 - 1.37 |
| Complete adult education/training |  |  | 1.26 | 0.27 | 0.279 | 0.83 - 1.92 |  | 1.17 | 0.26 | 0.473 | 0.76 - 1.80 |
| **Social capital** |  |  |  |  |  |  |  |  |  |  |  |
| Married (*ref*: Not) |  |  | 0.85 | 0.18 | 0.459 | 0.56 - 1.30 |  | 0.80 | 0.18 | 0.306 | 0.51 - 1.23 |
| Volunteer (*ref*: Not) |  |  | 1.70 | 0.35 | 0.010 | 1.13 - 2.55 |  | 1.64 | 0.35 | 0.019 | 1.08 - 2.48 |
| **Financial capital** |  |  |  |  |  |  |  |  |  |  |  |
| Income |  |  | 1.05 | 0.11 | 0.641 | 0.85 - 1.30 |  | 1.10 | 0.12 | 0.410 | 0.88 - 1.37 |
| Assets |  |  | 0.92 | 0.06 | 0.209 | 0.80 - 1.05 |  | 0.90 | 0.07 | 0.147 | 0.78 - 1.04 |
| **Personal preferences and values** |  |  |  |  |  |  |  |  |  |  |  |
| Startup reason: (*ref:* Work for oneself) |  |  |  |  |  |  |  |  |  |  |  |
| Make money |  |  | 0.51 | 0.13 | 0.009 | 0.31 - 0.84 |  | 0.51 | 0.13 | 0.007 | 0.31 - 0.83 |
| Meet social challenge, help others |  |  | 0.56 | 0.16 | 0.043 | 0.32 - 0.98 |  | 0.57 | 0.16 | 0.048 | 0.32 - 0.99 |
| Something else/Don’t know |  |  | 0.05 | 0.02 | 0.000 | 0.02 - 0.11 |  | - | - | - | - |
| Meaning of work: Personal |  |  | 1.03 | 0.04 | 0.466 | 0.95 - 1.11 |  | 1.03 | 0.04 | 0.448 | 0.95 - 1.12 |
| Social |  |  | 1.04 | 0.03 | 0.179 | 0.98 - 1.10 |  | 1.04 | 0.03 | 0.187 | 0.98 - 1.10 |
| Financial |  |  | 0.95 | 0.03 | 0.129 | 0.88 - 1.02 |  | 0.95 | 0.04 | 0.217 | 0.88 - 1.03 |
| Generativity |  |  | 1.05 | 0.05 | 0.262 | 0.96 - 1.16 |  | 1.05 | 0.05 | 0.317 | 0.95 - 1.16 |
| Constant |  |  | 56.72 | 77.86 | 0.003 | 3.83 - 839.00 |  | 56.67 | 80.81 | 0.005 | 3.45 - 930.18 |

*Note*. The binary dependent variable included “very interested” or “somewhat interested” = 1 and “not too interested” and “not at all interested” = 0; “All respondents” indicates all sample respondents, whereas “Without ‘something else/don’t know’” indicates that any respondent who reported that as a startup reason was excluded from the model; *SUE* = seemingly unrelated estimation results, indicating differences between the parameters of both groups with *p* < .05 indicated by *; *aOR* = adjusted odds ratio; *SE* = linearized standard error; *CI* = confidence interval.
