# Supplementary material for: The diversity of interest in later-life entrepreneurship: Results from a nationally representative survey of Americans aged 50 to 70
Source: PLoS One. 2019 Jun 5;14(6):e0217971. doi: 10.1371/journal.pone.0217971 (PMC6550427; doi:10.1371/journal.pone.0217971)
Supplement: S8 Table — (DOCX) [file pone.0217971.s008.docx]

**S8 Table. Multivariable Partial Proportional Odds Model Using the Autofit Feature in *Gologit2***

|  |  | **A** | | | |  | **B** | | | |  | **C** | | | |
| --- | --- | --- | --- | --- | --- | --- | --- | --- | --- | --- | --- | --- | --- | --- | --- |
|  |  | **aOR** | **SE** | **p** | **95% CI** |  | **aOR** | **SE** | **p** | **95% CI** |  | **aOR** | **SE** | **p** | **95% CI** |
| **Demographics** |  |  |  |  |  |  |  |  |  |  |  |  |  |  |  |
| Age |  | 0.92 | 0.02 | 0.000 | 0.89 - 0.95 |  |  |  |  |  |  |  |  |  |  |
| Gender |  | 0.65 | 0.11 | 0.009 | 0.48 - 0.90 |  |  |  |  |  |  |  |  |  |  |
| Race (*ref*: White, not Hispanic) |  |  |  |  |  |  |  |  |  |  |  |  |  |  |  |
| Black, not Hispanic |  | 2.35 | 0.65 | 0.002 | 1.36 - 4.05 |  |  |  |  |  |  |  |  |  |  |
| All other races |  | 0.49 | 0.16 | 0.026 | 0.26 - 0.92 |  | 1.71 | 0.51 | 0.076 | 0.95 - 3.08 |  | 1.66 | 0.65 | 0.196 | 0.77 - 3.58 |
| Rural (*ref*: Urban) |  | 0.80 | 0.16 | 0.258 | 0.55 - 1.18 |  | 1.25 | 0.24 | 0.246 | 0.86 - 1.81 |  | 0.88 | 0.25 | 0.644 | 0.51 - 1.52 |
| Work status (*ref*: Working for pay) |  |  |  |  |  |  |  |  |  |  |  |  |  |  |  |
| Self-employed |  | 1.89 | 0.80 | 0.132 | 0.82 - 4.32 |  | 1.93 | 0.61 | 0.038 | 1.04 - 3.58 |  | 4.13 | 1.32 | 0.000 | 2.21 - 7.73 |
| Retired |  | 0.49 | 0.13 | 0.007 | 0.30 - 0.82 |  | 0.85 | 0.23 | 0.549 | 0.50 - 1.44 |  | 0.94 | 0.35 | 0.868 | 0.45 - 1.97 |
| Disabled |  | 1.09 | 0.32 | 0.767 | 0.61 - 1.95 |  |  |  |  |  |  |  |  |  |  |
| Unemployed |  | 1.13 | 0.38 | 0.717 | 0.59 - 2.18 |  |  |  |  |  |  |  |  |  |  |
| Others |  | 0.79 | 0.21 | 0.379 | 0.46 - 1.34 |  |  |  |  |  |  |  |  |  |  |
| **Human capital** |  |  |  |  |  |  |  |  |  |  |  |  |  |  |  |
| Education (*ref*: High school or less) |  |  |  |  |  |  |  |  |  |  |  |  |  |  |  |
| Associate’s degree |  | 1.94 | 0.44 | 0.004 | 1.24 - 3.05 |  | 0.91 | 0.21 | 0.681 | 0.58 - 1.43 |  | 0.79 | 0.24 | 0.433 | 0.43 - 1.43 |
| Bachelor’s degree |  | 1.40 | 0.34 | 0.171 | 0.87 - 2.25 |  |  |  |  |  |  |  |  |  |  |
| Master’s degree and above |  | 1.73 | 0.59 | 0.107 | 0.89 - 3.37 |  | 1.01 | 0.34 | 0.981 | 0.52 - 1.96 |  | 0.70 | 0.29 | 0.391 | 0.31 - 1.59 |
| Health |  | 1.11 | 0.09 | 0.186 | 0.95 - 1.31 |  |  |  |  |  |  |  |  |  |  |
| Complete adult education/training |  | 1.29 | 0.23 | 0.144 | 0.92 - 1.83 |  |  |  |  |  |  |  |  |  |  |
| **Social capital** |  |  |  |  |  |  |  |  |  |  |  |  |  |  |  |
| Married (*ref*: Not) |  | 0.77 | 0.14 | 0.156 | 0.54 - 1.10 |  |  |  |  |  |  |  |  |  |  |
| Volunteer (*ref*: Not) |  | 1.56 | 0.25 | 0.006 | 1.14 - 2.15 |  |  |  |  |  |  |  |  |  |  |
| **Financial capital** |  |  |  |  |  |  |  |  |  |  |  |  |  |  |  |
| Income |  | 1.04 | 0.09 | 0.644 | 0.87 - 1.24 |  |  |  |  |  |  |  |  |  |  |
| Assets |  | 0.97 | 0.05 | 0.623 | 0.87 - 1.08 |  |  |  |  |  |  |  |  |  |  |
| **Personal preferences and values** |  |  |  |  |  |  |  |  |  |  |  |  |  |  |  |
| Startup reason: (*ref:* Work for oneself) |  |  |  |  |  |  |  |  |  |  |  |  |  |  |  |
| Make money |  | 0.56 | 0.12 | 0.008 | 0.37 - 0.86 |  |  |  |  |  |  |  |  |  |  |
| Meet social challenge, help others |  | 0.86 | 0.25 | 0.606 | 0.49 - 1.51 |  | 0.61 | 0.15 | 0.049 | 0.38 - 1.00 |  | 0.35 | 0.11 | 0.001 | 0.19 - 0.65 |
| Something else/Don’t know |  | 0.12 | 0.04 | 0.000 | 0.06 - 0.21 |  | 0.04 | 0.02 | 0.000 | 0.02 - 0.10 |  | 0.02 | 0.02 | 0.000 | 0.00 - 0.10 |
| Meaning of work: Personal |  | 0.97 | 0.03 | 0.397 | 0.90 - 1.04 |  | 1.03 | 0.04 | 0.419 | 0.96 - 1.11 |  | 1.12 | 0.05 | 0.021 | 1.02 - 1.23 |
| Social |  | 1.05 | 0.03 | 0.067 | 1.00 - 1.10 |  |  |  |  |  |  |  |  |  |  |
| Financial |  | 0.95 | 0.03 | 0.108 | 0.90 - 1.01 |  |  |  |  |  |  |  |  |  |  |
| Generativity |  | 1.08 | 0.05 | 0.099 | 0.99 - 1.17 |  |  |  |  |  |  |  |  |  |  |
| Constant |  | 165.73 | 188.90 | 0.000 | 17.70 - 1,551.73 |  | 17.94 | 21.31 | 0.015 | 1.74 - 184.58 |  | 0.75 | 0.95 | 0.818 | 0.06 - 9.13 |

*Note*. *Gologit2* is a user-written program for Stata created by Richard Williams (see reference numbers 61 and 64 for more information). When the parallel lines assumption is violated at the *p* < .05 level, the coefficients for the series of cumulative logit models are provided to aid in comparison: a = not very much or more interested vs. not at all interested; b = somewhat or more interested vs not very much or less interested; c = very interested vs. less than very interested.
